# Recommandations nutritionnelles pratiques avec exemples de menus pour personnes vivant avec le VIH/SIDA en Afrique Noire

**Published:** 2008-07-03

**Authors:** Estelle Anaëlle Nguewo, Gertrud Winkler

**Affiliations:** 1 Université de Sigmaringen, Allemagne

**Keywords:** VIH, Nutrition, Alimentation, Afrique, SIDA

## Abstract

L’infection par le VIH (Virus de l’Immunodéficience Humaine) est une infection chronique incurable. L’infection au VIH cause jusqu’à présent des millions de morts et entrave l’économie des pays sous développés ou en voie de développement, en l’occurrence les pays d’Afrique subsaharienne. Le but principal de la thérapie nutritionnelle en cas d’infection par le VIH est d’assurer au patient un poids normal et une alimentation appropriée couvrant ses besoins nutritionnels pendant les différentes phases de l’infection. Nous proposons des recommandations alimentaires détaillées quant aux apports recommandés en nutriments (macro- et micronutriments) pour les personnes vivant avec le VIH/SIDA. La nutrition joue en général un rôle très important dans le fonctionnement optimal du système immunitaire. Puisque l’infection par le VIH/SIDA est une maladie du système immunitaire, elle influence de plusieurs manières l’état nutritionnel du patient. Une nutrition hyper calorifique et hyper protéinée saine, variée et adaptée aux besoins de l’organisme est une condition indispensable pour rester longtemps en forme en cas d’infection par le VIH/SIDA. Elle permet en outre de garder un poids normal pendant la phase asymptomatique de l’infection et d’augmenter son poids pendant la phase symptomatique. Ceci a pour but de freiner l’évolution de l’infection vers le stade SIDA. Les personnes atteintes par le VIH/SIDA devraient en plus avoir une bonne hygiène de vie et exercer régulièrement une activité sportive modérée. Elles devraient mettre l’accent sur leur protection contre toute intoxication alimentaire et sur le renforcement de leur système immunitaire. Il est en outre important en plus du besoin en nutriments des personnes atteintes par le VIH/SIDA, de tenir compte de leur situation financière et culturelle, car en Afrique il existe sur ce point une très grande différence dans la population. C’est pourquoi, aussi à cause des différentes causes de la perte involontaire de poids et des différents goûts alimentaires des personnes, il est recommandé que les conseils et le suivi nutritionnel du patient soient adaptés à l’individu.

## Introduction

L’infection par le VIH (Virus de l’Immunodéficience Humaine) est une infection chronique incurable. Elle cause jusqu’à présent des millions de morts et entrave l’économie des pays sous développés ou en voie de développement, en l’occurrence les pays d’Afrique subsaharienne. C’est pourquoi il est important d’endiguer l’expansion de l’infection aussi bien que d’atténuer les maux et augmenter la qualité de vie des personnes déjà souffrantes. Une contribution à cela est une alimentation appropriée à la phase de l’infection.

Les articles suivants proposent des recommandations alimentaires détaillées quant aux apports recommandés en nutriments (macro- et micronutriments) pour les personnes vivant avec le VIH/SIDA. Ce travail propose également des conseils pratiques en cas de problèmes alimentation et des exemples de menus adaptés au contexte africain. Les conseils nutritionnels que nous proposons s’adressent à tous ceux qui ont le souci de se nourrir sainement et ne concernent pas uniquement les personnes vivantes avec le VIH/SIDA,

### Description de l’évolution de l’infection

L’infection par le VIH affaiblit le système immunitaire en détruisant les cellules responsables de la réaction immunitaire (cellules CD4 ou T4) et en se multipliant à l’intérieur de celles-ci. Ces dernières ne peuvent plus remplir leurs fonctions à savoir assurer la défense de l’organisme contre les agents pathogènes [1].

Au cours de l’évolution de l’infection par le VIH, on distingue deux stades principaux: le stade asymptomatique et le stade symptomatique.

Le stade asymptomatique comme son nom l’indique est sans symptôme. Juste après la contamination apparaît, à peine perceptible, des signes similaires à ceux d’une grippe (c’est l’infection primaire). Le corps infecté développe des anticorps qui sont en règle générale détectables dès la douzième semaine. Ce stade asymptomatique varie d’un individu à l’autre, peut durer des années et est influencer entre autre par l’environnement sanitaire et nutritionnel général du sujet [2]. Plus la charge virale est élevée, plus rapidement le système immunitaire est compromis. L’affaiblissement du system immunitaire favorise le développent des affections opportunistes, aussi bien infectieuses que cancéreuses et la progression vers le SIDA et éventuellement la mort du patient [1, 3]. La progression de l’infection initiale à l’effondrement total du système immunitaire varie d’un patient à l’autre [4].

### VIH/SIDA et nutrition

#### Influence de l’infection sur la nutrition

Les interactions entre la nutrition et l’infection par le VIH sont multiples ( Figure 1 ), et en ce qui concerne les micronutriments, sont en partie contradictoires.

Par exemple une carence en Vitamine A ou en Sélénium favorise le passage du VIH à travers les parois muqueuses du tractus uro-génital en altérant l’intégrité des tissus épithéliaux [5, 6, 7] .

Une nutrition saine et équilibrée à long terme assure à l’organisme infecté une résistance optimale aux agents pathogènes. Elle permet par exemple de compenser les pertes énergétiques dues à l’infection et permet de maintenir les organes en bon état de fonctionnement. C’est pour cela qu’il est indispensable que l’organisme infecté reçoive suffisamment de nutriments qui renforcent la fonction immunitaire et améliorent le fonctionnement des cellules. Il s’agit comme micronutriments, de la vitamine A, E, Beta-Carotène, Thiamine (B _1_ ), Riboflavine (B _2_ ), Niacine (B _3_ ), acide pantothénique (B _5_ ), Pyridoxine (B _6_ ), Biotine (H ou B _8_ ) acide folique (B _9_ ), Cobalamine (B _12_ ) et des oligo-éléments Zinc, Sélénium, Cuivre, Fer et Manganèse [2, 8, 9, 10]

La malnutrition et la perte involontaire de poids (Wasting-syndrom) sont les principales conséquences de l’infection à VIH. Celles-ci sont soit directement dues à l’infection soit sont la conséquence des multiples traitements pris pour le traitement de la maladie ou des affections opportunistes [11].

Les causes les plus fréquentes de malnutrition chez les personnes infectées par le VIH sont [9]:

- L’augmentation des besoins énergétiques et nutritifs due en général aux poussées de fièvre et aux infections opportunistes
- La diminution des apports alimentaires
- Les troubles d’absorption des aliments et nutriments
- Les perturbations métaboliques dues aux troubles du système immunitaire, nerveux ou gastrique et aux infections opportunistes
- Les problèmes psychiques et sociaux comme par exemple la stigmatisation, la discrimination, l’isolation, la dépression et la pauvreté

La perte de poids involontaire est caractérisée par une diminution de la masse musculaire corporelle et non automatiquement de la masse graisseuse. Ceci se manifeste par une fatigue extrême et une altération de l’état générale et une détérioration de la qualité et de l’espérance de vie du patient. [8; 11, 12].

#### Influence des médicaments sur la nutrition

Le but principal de la thérapie antirétrovirale est d’entraver la multiplication du VIH, de restaurer le système immunitaire et par conséquence de retarder l’apparition des infections opportunistes et de l’évolution vers le SIDA et d’améliorer la qualité de vie du patient [14].

Ces médicaments peuvent cependant influencer de façon considérable la nutrition des personnes infectées et les troubles gastro-intestinaux dus à la prise des médicaments antirétroviraux [9] sont fréquents chez les patients sous thérapie VIH.

Les principaux effets secondaires des médicaments antirétroviraux utilisés de nos jours sont la nausée, les vomissements, le manque d’appétit et la diarrhée (Tableau 1).

En outre les médicaments utilisés en Afrique contre les affections opportunistes ont également de par leurs effets secondaires une influence notable sur la nutrition du patient. Le tableau 2 présente ces médicaments et leurs effets secondaires.

## Etat des lieux

### Recommandations nutritionnelles

#### But de la thérapie nutritionnelle

Le but principal de la thérapie nutritionnelle en cas d’infection par le VIH est d’assurer au patient un poids normal et une alimentation appropriée couvrant ses besoins nutritionnels pendant les différentes phases de l’infection. L’institut allemand de la médecine alimentaire a par établi les objectifs des en vue de réduire les doléances des patients en ce qui concerne leur nutrition, le maintient de leur force physique afin de préserver leur indépendance pour leurs activités quotidiennes, et de réduire voire empêcher leurs séjours à l’hôpital (Tableau 3) [17]:

### Recommandations quant á l’approvisionnement en nutriments en cas d’infection par le VIH

Les recommandations actuelles quant à l’apport en nutriments en cas d’infection par le VIH différent de celles des personnes saines surtout en ce qui concerne les éléments suivants: énergie, protéines, vitamines A, B _1_ , B _2_ , B _3_ , B _6_ , B _12_ , C et E ainsi que la béta carotène et l’oligo-élément Zinc (Tableau 4).

Les besoins énergétiques d’un patient atteint de VIH augmentent en général de 10 à 15% et les besoins protéiniques de 50 á 100%, particulièrement durant la phase symptomatique [18, 19]. Leurs besoins en protéines varient en général de 1,2 à 2,0 g/kg p.c./j (gramme par kilogramme de poids corporel par jour), et peut atteindre 3,0 g/kg p.c./j durant la phase symptomatique [20, 20] *.* Les besoins énergétiques d’une femme enceinte infectée par le VIH augmentent eux jusqu’á 20%0 voire 30% [22]

Les hydrates de carbones quant à eux devraient couvrir environ 60 % de l’apport énergétique total et l’apport quotidien en matières grasses devrait être compris entre 1,2 et 1,8 g/kg p.c. [20, 23].

Plus la perte involontaire de poids est prononcée, plus riche devrait être l’alimentation en énergie. Cette énergie devrait cependant provenir principalement des protéines et des hydrates de carbones [8]. Il est généralement recommandé de varier l’approvisionnement en nutriments en fonction du stade de la maladie, la constitution générale du malade et de la thérapie antirétrovirale afin par exemple de compenser les pertes nutritives dues aux effets secondaires des médicaments [8; 11]. En outre il est très important pour les personnes infectées par le VIH d’avoir une alimentation saine, équilibrée, variée et adaptée aux besoins de leur organisme.

Si le poids du patient ne réussit pas à être stabilisé par une alimentation naturelle, la motivation et l’amélioration de son environnement social, on devrait faire appel à une alimentation artificielle (parentérale et entérale) ainsi qu’aux suppléments alimentaires [8, 11].

### Thérapie nutritionnelle adaptée à la situation alimentaire en Afrique noire

Les protéines de haute qualité biologique ne devraient pas manquer dans l’alimentation quotidienne des personnes infectées par le VIH [8, 9, 20, 21] et les protéines végétales devraient être préférées à celles animales. En plus de la viande, du poisson et des produits laitiers, il existe pour un approvisionnement adapté en protéines des combinaisons peu coûteuses d’aliments végétaux ayant une qualité biologique élevée (par exemple ragout de haricots rouges avec sauce d’arachides et graines de mais) ainsi que le soja, qui en plus de flavonoïdes est riche en acides gras insaturés. Il est recommandé de manger du poisson riche en acides gras Omega-3 et Omega-6 comme par exemple le hareng, le maquereau ou le thon, car ces acides gras jouent un rôle important dans le renforcement du système immunitaire [24].

Les aliments énergétiques tels que les céréales (de préférence complètes), les plantes racines de toutes sortes (manioc, macabo, taro, patate…) et les fruits féculents (banane, banane plantain…) devraient être abondamment consommés.

Les recommandations générales de l’OMS “5 portions de fruits et légumes par jour” couvrent les besoins journaliers en antioxydants et peut être repartir comme suit: 2 portions de fruits et trois portions de légumes y compris les crudités, les légumes cuits et les laitues. Les jus d’orange et de pamplemousse sont particulièrement riches en antioxydants [25].

Comme huile de cuisson, les huiles végétales (par example. l’huile de soja, d’arachides…) sont les plus conseillées car elles présentent une meilleure composition d’acides gras (taux élevé d’acides gras insaturés) que les graisses animales.

### Recommandations d’hygiène

En plus des recommandations d’approvisionnement en macro et micronutriments, il est important pour toute personne en général et les personnes atteintes de VIH/SIDA en particulier d’observer les règles d’hygiène alimentaire.

Concernant la cuisine et les aliments [13, 26]:

- De façon idéale il serait préférable pour la nutrition des personnes atteintes de VIH/SIDA de ne choisir que des aliments frais.
- Les aliments ne devraient pas avoir de longues voies de transport, de préférence les acheter directement chez le producteur ou au marché au petit matin quand ils sont encore bien frais.
- Les aliments surtout ceux mangés crus (fruits, crudités) devraient avant leur utilisation être bien lavés à l’eau propre filtrée (eau potable). On peut utiliser de l’eau de javel (une petite cuillère d’eau de javel pour un litre d’eau) pour laver les aliments.
- Les viandes ainsi que les poissons doivent être cuits à point.
- Les personnes atteintes de VIH/SIDA devraient éviter de manger des repas qui ont passé la nuit afin d’éviter d’éventuelles intoxications alimentaires.
- Les restes de repas doivent être bien couverts et si possible gardés dans un réfrigérateur ou dans un endroit frais bien propre.
- Les repas ne doivent pas être maintenus chauds pendant longtemps (perte de nutriments sensibles à la chaleur).
- Pendant la préparation des aliments pour la cuisson, les viandes et les poissons doivent être séparés des aliments prêts à être consommés.
- Les aliments devraient être apprêtés dans un environnement propre. Les torchons de cuisine eux aussi doivent être propres et doivent être régulièrement changés. La poubelle doit être constamment hermétiquement fermée et placée loin des aliments et du foyer de cuisson.
- Il est important que les règles personnelles d’hygiène soient observées tant par la personne qui cuisine que par celles qui mangent. Lavage des mains après toilettes et quand le repas est mangé à la main.
- Les repas vendus au bord de la route ne sont pas conseillés à cause d’un manque d’hygiène possible.

#### Concernant l’eau:

L’approvisionnement en eau potable est un problème dans plusieurs pays africains. Surtout en campagne où peu de ménages ont accès a de l’eau du robinet. L’eau insalubre est l’une des principales sources d’infections bactériennes en Afrique [27]. C’est pourquoi les personnes infectées par le VIH doivent s’assurer de la propreté et la pureté de l’eau qu’elles boivent. Cette eau, qu’elle soit du robinet ou du puits doit être bouillie ou filtrée. En plus il est à noter que :

- Le puits doit toujours être propre et hermétiquement fermé.
- Ce n’est qu’après avoir bouilli de l’eau pendant au moins cinq minutes que la majorité des micro-organismes nocifs qui peuvent s’y trouver est éliminée [24]. Malheureusement les impuretés chimiques elles ne sont pas éliminées ainsi.
- La filtration quant à elle permet d’éliminer les impuretés chimiques mais pas les micro-organismes nocifs. (Vous trouverez des instructions pour la fabrication d’un filtre dans Epstein [24].)
- C’est pourquoi une combinaison des deux méthodes (bouillir de l’eau, laissé refroidir et filtrer) est vivement recommandée.

## Exemples de menus (adaptes aux habitudes alimentaires en Afrique noire) pour personnes infectées par le VIH/SIDA

Le tableau 5 donne le rôle et la source locale de quelques micronutriments importants pour une alimentation saine [29, 30,31]. Le tableau 6 donne des exemples de menus équilibrés à base d’aliments locaux. Les tableaux 7a et 7b quant à eux donne des propositions de menus journaliers détaillés et mis sur pied à l’aide d’un programme de nutrition (PRODI).

## Conclusion

La nutrition joue en général un rôle très important dans le fonctionnement optimal du système immunitaire. Puisque l’infection par le VIH/SIDA est une maladie du système immunitaire, elle influence de plusieurs manières l’état nutritionnel du patient. Une nutrition hyper calorifique et hyper protéinée saine, variée et adaptée aux besoins de l’organisme est une condition indispensable pour rester longtemps en forme en cas d’infection par le VIH/SIDA. Elle permet en outre de garder un poids normal pendant la phase asymptomatique de l’infection et d’augmenter son poids pendant la phase symptomatique. Ceci a pour but de freiner l’évolution de l’infection vers le stade SIDA. Les personnes atteintes par le VIH/SIDA devraient en plus avoir une bonne hygiène de vie et exercer régulièrement une activité sportive modérée. Elles devraient mettre l’accent sur leur protection contre toute intoxication alimentaire et sur le renforcement de leur système immunitaire.

Il est en outre important en plus du besoin en nutriments des personnes atteintes par le VIH/SIDA, de tenir compte de leur situation financière et culturelle, car en Afrique il existe sur ce point une très grande différence dans la population. C’est pourquoi, aussi à cause des différentes causes de la perte involontaire de poids et des différents goûts alimentaires des personnes, il est recommandé que les conseils et le suivi nutritionnel du patient soient adaptés à l’individu.

## Tableaux et figures

- Tableau 1: Exemples de quelques médicaments antirétroviraux utilisés en Afrique avec leurs influences sur la nutrition des patients
- Tableau 2: Exemples de médicaments utilisés et leur influence sur la nutrition : Objectifs nutritionnels de l’institut allemand de la médecine alimentaire pour les personnes vivant avec le VIH/SIDA.
- Tableau 4: Comparaisons de Recommandations journalières de quelques nutriments pour personnes saines (D-A-CH (Allemagne-Autriche-Suisse) 2000) et pour personnes infectées par le VIH.
- Tableau 5: Nutriments importants pour une alimentation saine, leurs fonctions dans l’organisme et leurs sources locales.
- Tableau 6: Propositions de menus (sans données de quantités) pour personnes vivant avec le VIH/SIDA en Afrique noire.
- Tableau 7a: Menu pour une femme adulte d’activité moyenne infectée par le VIH/SIDA en phase asymptomatique.
- Tableau 7b: Menu pour une femme adulte d’activité moyenne infectée par le VIH/SIDA en phase symptomatique.
-
  **Figure 1:**
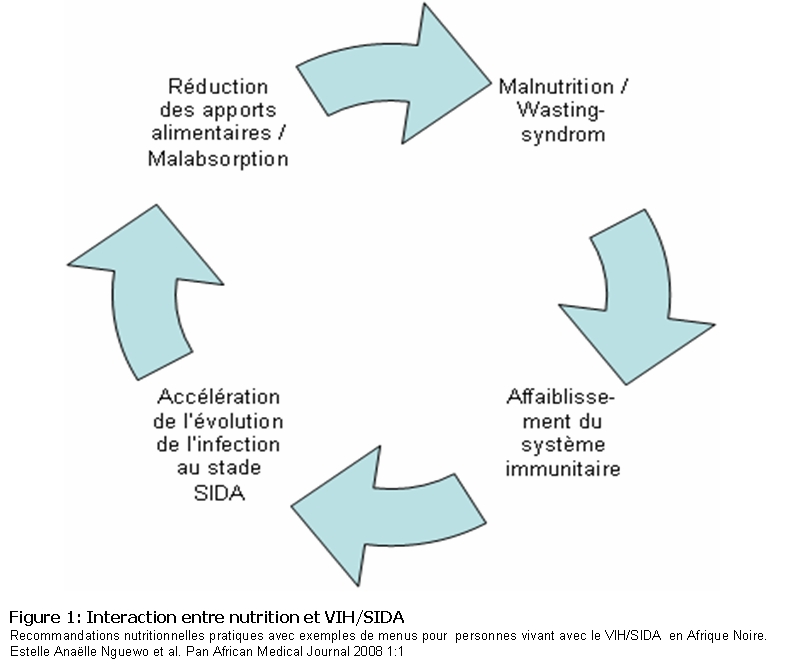
Interaction entre nutrition et VIH/SIDA

## Figures and Tables

**Tableau 1:** Exemples de quelques médicaments antirétroviraux utilisés en Afrique avec leurs influences sur la nutrition des patients.

** Médicaments **	** Effets secondaires influençant la nutrition **	** Recommandations de prise (capsules/comprimés par jour) **
Emtriva® (Emtricitabine)	Nausée, diarrhée	1 x 200 mg Avec ou sans aliments
Retrovir® (Zidovudine)	Nausée, douleur d’estomac, vomissement	2 x 250 mg 2 x 300 mg Avec de l’eau propre, avec ou sans aliments
Videx® (Didanosine)	Nausée, diarrhée	2 x 200 mg 2 x 100 mg, á jeun
Ziagen® (Abacavir)	Nausée, vomissement, fièvre	2 x 300 mg, avec ou sans aliments
Viread® (Tenofovir)	Diarrhée, nausée, vomissement	1 x 300 mg avec aliments gras
Crixivan® (Indinavir)	Calcul rénal (boire beaucoup: >2l/jour) troubles du métabolisme des lipides	3 x 800 mg, pas avec des aliments riches en graisses et en protéines
Norvir ® (Ritonavir)	Nausée, paresthésie orale, diarrhée, perte de goût	2 x 600 mg, pendant les repas
Viracept® (Nelfinavir)	Diarrhée	2 x 1250 mg, pendant les repas

**Tableau 2:** Exemples de médicaments utilisés et leur influence sur la nutrition.

** Médicament **	** Indication **	** Effets secondaires possibles **	** Recommandations **
Chloroquine	Paludisme	Douleur d’estomac, diarrhée, perte d’appétit, nausée, vomissement.	Avec aliments. Non recommandée pour les femmes qui allaitent.
Fluconazole	Candidose buccale	Nausée, vomissement, diarrhée.	Avec aliments. Peut être utilisé pendant l’allaitement.
Isoniazide	Tuberculose	Insociabilité possible avec des aliments comme. banane, bière, avocat, boissons avec caféine, chocolat, saucisse, poisson fumé, levure et yaourt. Peut interférer avec le métabolisme de la vitamine B_6_ et par conséquent exiger la prise de la vitamine B_6_.	A jeun, au moins 1–2 heures avant le repas
Nystatine	Muguet buccal	(rarement) Diarrhée, vomissement, nausée	Avec aliments
Quinine	Paludisme	Douleur abdominale ou d’estomac, diarrhée, nausée, vomissement, baisse du taux de sucre dans le sang.	Avec aliments
Rifampicine	Tuberculose	Nausée, vomissement, diarrhée, perte d’appétit.	A jeun, au moins 1–2 heures avant le repas
Sulfadoxine et Pyrimethamine (Fansidar)	Paludisme	Nausée, vomissement.	Avec aliments. Non recommandé si déficience en folate et pour les femmes qui allaitent. Boire beaucoup d’eau propre (filtrée et bouillie).
Sulfamides: Sulfamethoxazole, Cotrimoxazole (Bactrim, Septra)	Pneumonie et Toxoplasmose	Nausée, vomissement, douleur abdominale	Avec aliments

**Table 3 :** Objectifs nutritionnels de l’institut allemand de la médecine alimentaire pour les personnes vivants avec le VIH/SIDA.

Maintien ou optimisation de l’état nutritionnel
Stabilisation du poids (éviter toute perte de poids ou tout sous-poids)
Maintien de la masse cellulaire corporelle
Diminution des handicaps fonctionnels dus à une dénutrition (faiblesse musculaire, fatigue, alitement, incapacité de travail)
Conservation et amélioration du bien-être subjectif
Augmentation de la tolérance d’une thérapie antirétrovirale
Atténuation des symptômes gastro-intestinaux de l’infection par le VIH
Prévention des déficits en vitamines et en sels minéraux
Evitement des aliments incompatibles (qui pour une raison ou une autre ne sont pas supportés par le patient)
Evitement d’aliments susceptibles de causer une infection ou une intoxication alimentaire
Instauration à temps (au bon moment) d’une alimentation artificielle

**Tableau 4:** Comparaisons de Recommandations journalières de quelques nutriments pour personnes saines (D-A-CH (Allemagne-Autriche-Suisse) Referenzwerten 2000) et pour personnes infectées par le VIH.

	**Recommandations journalières**		

**Nutriments**	**Valeurs référentielles OMS/FAO**	**Valeurs référentielles D-A-CH(Allemagne-Autriche-Suisse) 2000**	**Patient atteint de VIH**
Energie	24 – 42 kcal/kg KG	25 – 35 kcal/kg PC	>25 – 35 kcal/kg PC^1^
Protéine	10 – 15 en%	0,8 g/kg PC	1,2 bis 2 g/kg PC
Hydrate de carbone	55 – 75 en%	55 en%^2^	2 – 5 g/kg KG (60en%^2^)
Matières grasses	15 – 30 en%	25 bis 30 en%^2^	1,2 – 1,8 g/kg P.C.^3^
Boissons	1,04 – 1,63 Liter	1,5 – 2 Litre	2 – 3 Litre
Vitamine A	O,5 – 0,85 mg Ä^4^	0,8 –1,5 mg Ä^4^	1,3 – 3,0 mg
Béta carotène		30 mg	30 – 50 mg
Vitamine E	7,5 – 10 mg Ä^5^	12 – 17 mg Ä^5^	30 – 300 mg
Vitamine C	45 – 70 mg	100 mg	200 – 500 mg
Vitamine B_1_ (Thiamine)	1,1 – 1,5 mg	1 – 1,3 mg	3,0 – 7,5 mg
Vitamine B_2_ (Riboflavine)	1,0 – 1,6 mg	1,2 – 1,5 mg	3,4 – 8,5 mg
Vitamine B_3_ (Niacine)	14 – 18 mg Ä^6^	13 – 17 mg Ä^6^	38 – 95 mg
Vitamine B_6_ (Pyridoxine)	1,3 – 2,0 mg	1,2 – 1,5 mg	4 – 10 mg^7^
Vitamine B_12_ (Cobalamine)	2,4 – 2,8 μg	3 μg	4 – 10 μg
Zinc	4,9 – 10 mg	7 – 10 μg	15 mg

P.C = Poids Corporel.

1 en cas de Wasting-Syndrom. Les matières grasses animales devraient cependant être évitées á cause du taux élevé en cholestérol.

2 en% = Pourcentage par rapport á l’énergie totale.

3 30 en% en cas de Wasting-Syndrom et provenant surtout des matières grasses végétales et des acides gras Omega-3/6.

4 Retinol mg-équivalent.

5 Tocopherol mg-équivalent.

6 Niacin mg-équivalent.

7 recommandé surtout pour patients atteints de VIH et qui souffrent de tuberculose et prennent de l’isoniazide.

**Tableau 5:** Nutriments importants pour une alimentation saine, leurs fonctions dans l’organisme et leurs sources locales.

**Micronutriments**	**Rôles dans l’organisme**	**Sources (aliments locaux)**
Vitamine A	Amélioration de l’efficacité du système immunitaire. Important pour la croissance des cellules et la santé des yeux et de la peau. Renforcement de l’intégrité des tissus épithéliaux.	Abats (foie, cœur, rognons…), poissons, produits laitiers, œufs, ail, légumes-feuilles vert foncé, patates douces, Luzerne, fruits et légumes jaunes ou oranges tels que papayes, oranges, potirons, carottes, patates douces…
Thiamine = Vitamine B_1_	Maintien de l’appétit et des fonctions du système nerveux. Important pour le métabolisme de l’énergie.	Céréales, haricots rouges, poissons, viande, poulet, œufs.
Riboflavine = Vitamine B_2_	Maintien de la santé, de la vision et de l’intégrité de la peau. Important pour le métabolisme de l’énergie.	Lait, yaourt, haricots rouges, poisson, viande, légumes-feuilles vertes, céréales complètes.
Niacine = Vitamine B_3_	Maintien de la santé et de l’intégrité de la peau, du système nerveux et de l’appareil digestif. Important pour le métabolisme de l’énergie.	Lait, œufs, arachides, céréales complètes, poisson, volaille, viande…
Pyridoxine = Vitamine B_6_	Facilitation du métabolisme et de l’absorption des matières grasses et des protéines. Important pour la fabrication des globules rouges.	Patates douces, mais, avocats, choux, noix, légumineuses, bananes, poisson, viande…
Acide folique = Vitamine B9	Travail de collaboration avec la vitamine B _12_ . Participation á la formation des globules rouges, á la division et la maturation des cellules.	noix, légumineuses, légumes verts, céréales complètes, avocats, oranges, œufs, poisson, abats…
Cobalamine = Vitamine B_12_	Action anti oxydante effective. Maintien des cellules nerveuses. Importante pour le développement de nouvelles cellules.	Produits fermentés, lait, œufs, fromage, crustacés, poisson, poulet, abats, viande rouge…
Acide ascorbique = Vitamine C	Affermissement du système immunitaire. Important pour l’absorption du fer et la croissance des os et des dents.	agrumes (citrons, oranges, pamplemousse…), tomates, Luzerne, pommes de terre, poivron, légumes-feuilles verts, goyaves…
Vitamine E	augmentation de la résistance contre les maladies. Important pour la protection des structures des cellules et le ralentissement du vieillissement. Action anti oxydante.	Noix, graines (soja, haricots blancs…) céréales complètes, légumes, légumes-feuilles, Luzerne, huiles végétales, jaune d’œuf, foie…
Fer	Transport de l’oxygène dans le sang Important pour l’élimination des anciens globules rouges,, la fabrication de nouveaux globules rouges ainsi que le fonctionnement des enzymes.	Légumes-feuilles vertes, crustacés, haricots rouges, lentilles, arachides, œufs, céréales complètes, abats (surtout foie et rognons), volaille, viande rouge…
Calcium	Renforcement des os et des dents. Important pour le bon fonctionnement du cœur et des muscles ainsi que pour la coagulation du sang et les défenses immunitaires.	Lait, Légumes-feuilles vertes, crevettes, poisson séché, haricots rouges, lentilles, poids, mil complet, gombo…
Zinc	Renforcement du système immunitaire. Facilitation de la digestion. Transport de la vitamine A. Important pour le développement des muscles.	Graines de citrouille (Pistaches), noix, lait, céréales complètes, jaune d’ œuf, ail, crustacés, légumes, légumineuses, mais, poisson, poulet, foie, viande…
Sélénium	Action anti oxydante Protection des cellules.	céréales complètes, lait, ail, oignons, tomates, carottes, haricots rouges, jaune d’ œuf, luzerne, crustacés, foie, œufs, viande…
Iode	Assurance du bon développement et fonctionnement correct du cerveau et du système nerveux.	poisson, fruits de mer, sel iodé…
Magnésium	Renforcement des muscles. Contribution à la croissance des os et au maintien des dents. Important pour le bon fonctionnement du système nerveux.	céréales complètes, légumes, fruits de mer, noix, graines, avocats…

**Tableau 7a :** Menu pour une femme adulte d’activité moyenne infectée par le VIH/SIDA en phase asymptomatique: environ 2200 kcal (9000 kJ), d’où 13 % proviennent des protéines, 30 % des matières grasses et 57 % des hydrates de carbone; apport adéquat en fibres, vitamines et sels minéraux. Calcul et optimisation des valeurs nutritives à l’aide du programme PRODI.

**Petit déjeuner**	**Snack**	**Repas de midi**	**Snack**	**Repas du soir**	**Snack**
**Pain à la purée d’avocat (de préférence du pain complet)** 100 g de pain 1 petit avocat mûr 3 tomates moyennes Sel et poivre 1 Tasse de Thé avec 2 morceaux de sucre	**Yaourt aux fruits** 250 g de yaourt nature entier 100 g d’Ananas 100 g de Papaye 1 cuillère à soupe de miel	**Légumes en feuilles à la sauce tomate.** (pour 4 personnes) 1 kg de légumes en feuilles découpés en petits morceaux 40 ml d’huile de soja 4 petits oignons 2 grosses gousses d’ail 8 tomates moyennes 40 g de condiments verts frais (persil) sel iodé poivre Complément: 600 g de plantains	250 ml de jus de fruits frais 1 grosse banane	**Omelette accompagnée de salade verte:** (pour 1 Personne) 2 petits œufs 1 cuillère à soupe d’huile de soja ou d’arachides 1 cuillère à soupe de différents condiments verts hachés sel iodé poivre 50 g de poivron 50 g de concombre 100 g de pain 5 g de margarine	1 Tasse de citronnelle avec 2 morceaux de sucre.
**Préparation:** Ecraser la chair de l’avocat (à l’aide d’une fourchette) dans un saladier après avoir enlevé le noyau et la pelure. Découper les tomates en quartiers et les ajouter á la purée d’avocat. Assaisonner avec le sel et le poivre. Tartiner le pain avec.	**Préparation:** Découper les fruits en petits morceaux et mélanger au yaourt. Sucrer avec le miel.	**Préparation:** faire revenir les oignons, l’ail et les tomates découpés en petits morceaux dans l’huile brièvement chauffé dans une poêle. Ajouter le reste des ingrédients et remuer le tout. Laisser cuire à feu doux et couvert pendant 20 mn. Servir les légumes avec du plantain cuit.		**Préparation:** battre les œufs avec les condiments verts, assaisonner de sel et de poivre. Chauffer brièvement l’huile dans une poêle et y verser les œufs battus. Laisser cuire des 2 côtés, servir sur le pain et garnir avec le poivron et le concombre découpés en morceaux. Pour la salade : 100 g de laitue 50 g de graines de mais cuits Mélanger 2 cuillères à soupe d’huile à 1 cuillère à soupe de vinaigre et un petit oignon découpé en petits morceaux, assaisonner de sel et de poivre et verser sur la salade.	
**Boire en plus au moins 750 ml d’eau potable propre (eau bouillie et filtrée) tout le long de la journée!**					

**Tableau 7b :** Menu pour une femme adulte d’activité moyenne infectée par le VIH/SIDA en phase symptomatique: environ 2400 kcal (10000 kJ), d’où 16,5 % proviennent des protéines, 28,5 % des matières grasses et 55 % des hydrates de carbone; apport adéquat en fibres, vitamines et sels minéraux

**Petit déjeuner**	**Snack**	**Repas de midi**	**Snack**	**Repas du soir**	**Snack**
90 g de pain tartiné avec de la pâte d’arachides 50 g de carottes 50 g de concombre 1 Tasse de lait demi-écrémé avec 2 morceaux de sucre	Graines de soja germées 1 Tasse de jus « vitaminé » **Jus vitaminé :** 1 cuillère à soupe de miel 1 jaune d’œuf biologique (Œuf du village) Jus de 2 grosses ou 3 oranges moyennes Jus d’un citron 2 carottes râpées Réduire le tout en purée et boire. Donne plus de vitamines et de force.	**Ratatouille:** (pour 4 personnes) 3 cuillères à soupe de jus de citron 500 g de filets de poisson 250 g d’aubergine 60 ml d’huile de soja 250 g de courgette 250 g de haricot vert 4 oignons moyens 2 poivrons 2 gousses d’ail 200 g de tomates 300 g de pâte de soja 300 g de riz complet	75 g de chips de plantains 50 g d’arachides grillées	**Salade de fruits** (pour 1 Personne) 50 g d’ananas 50 g de papaye 1 morceau de pastèque 1 cuillère à soupe de miel 90 g de pain beurré avec 10 g de margarine	1 Tasse de thé vert avec 2 morceaux de sucre
**Boire en plus au moins 1250 ml d’eau potable propre (eau bouillie et filtrée) tout le long de la journée!**		**Préparation:** arroser le poisson du jus de citron et le saler. Le cuire avec un peu d’eau pendant 10 à 15 mn dans une casserole fermée. Découper les légumes oignons et ail en petit morceaux. Faire revenir d’abord les courgettes dans la moitie de l’huile. Les retirer et les laisser égoutter. Verser le reste d’huile dans la poêle, y faire revenir les oignons pendant 2 mn. Ajouter le haricot vert, laisser cuire 3 mn, ensuite ajouter les poivrons, tomates, pâte de soja et ail et laisser mijoter pendant 10 mn. Y ajouter l’aubergine et les courgettes, laisser cuire de nouveau 3 mn. Assaisonner selon son goût avec sel et poivre. Servir les légumes avec le poisson et le riz complet cuit à l’étuvée.		**Préparation:** Découper les fruits en petits morceaux et les mélanger dans un saladier. Arroser d’une giclée de jus de citron frais. Manger accompagné du pain beurré.	

## References

[1] Pezzutto A, Ulrichs T, Burmester GR (2007). Taschenatlas der Immunologie. Grundlagen, Labor, Klinik. 2. Auflage.

[2] Rajabiun S, Cogill B, Seumo-Fosso E (2002). VIH/SIDA: Un guide pour les soins et le soutien nutritionnel. FANTA (Hg).

[3] Kaplan J, Hu D, Holmes K, Jaffe H, Masur H, De Cock K (1996). Preventing opportunistic infections in human immunodeficiency virus-infected persons: implication for the developing world. Am. J. Trop. Med. Hyg..

[4] Kamps BS, Hoffmann C, Kamps BS (2004). Epidemie, Übertragungswege, natürlicher Verlauf. HIV.NET.

[5] Baeten JM, Mostad S, Hughes M, Overbaugh J, Bankson D, Metaliya K, Ndinya-Achola J, Bwayo J, Kreiss J (2001). Selenium deficiency is associated with shedding of HIV-1-infected cells in the female genital tract. J. AIDS.

[6] Semba R (1998). The role of vitamin A and related retinoids in immune function. Nutr. Rev..

[7] De Luca L, Darwiche N, Celli G, Kosa K, Jones C, Ross S, Chen L (1994). Vitamin A in epithelial differentiation and skin carcinogenesis. Nutr. Rev..

[8] Kasper H (2004). Ernährungsmedizin und Diätetik. 10. neubearbeitete Auflage.

[9] Schwenk A, Biesalski HK (2004). Ernährung bei HIV-Infektion und AIDS. 3. erweiterte Auflage.

[10] Baum MK, Shor-Posner G, Lu Y, Rosner B, Sauberlich H, Fletcher M, Szapocznik J, Eisdorfer C, Buring J, Hennekens C (1995). Micronutrients and HIV-1 disease progression. AIDS.

[11] Schwenk A, Schauder P, Ollenschläger G (2003). Mangelernährung und Stoffwechselstörungen bei HIV-Infektion. 2, Auflage.

[12] Kotler D, Tierny A, Wang J, Pierson RJ (1989). magnitude of body-cell-mass depletion and the timing of death from wasting in AIDS. Am. J. Clin. Nutr..

[13] Nguewo EA (2007). Ernährungsempfehlungen für HIV-infizierte Menschen – Entwicklung eines praktischen Leitfadens für Kamerun. Mémoire à l’université d’Albstadt-Sigmaringen.

[14] WHO (2005). AIDS treatment, nutrition and food supplements. WHO fact sheet, 30.

[15] Das Kompetenznetz der Medizin HIV/AIDS (KompNet HIV/AIDS). http://www.kompetenznetz-hiv.de/./index.htm.

[16] Arzneimittel zur antiretroviralen Therapie der HIV-Infektion. http://www.aidsfinder.org/main/af_53.htm#5.3.1.

[17] Ockenga J, Stüttmann U, Schwenk A (2003). Wasting bei Infektionskrankheiten. Aktuel Ernähr Med.

[18] James WPT, Schofield EC (1990). Human Energy Requirements: A manual for planners and nutritionists.

[19] WHO (1985). Energy and Protein Requirements. Technical Report Series 724.

[20] Berger DS, Miller T, Gorbach G (1999). Enteral and parenteral support. Nutritional aspects of HIV infection.

[21] Woods M, Miller T, Gorbach G (1999). Dietary recommendation for the HIV/AIDS patient. Nutritional aspects of HIV infection.

[22] Nutritional Guidelines for Care and support for people living with HIV/AIDS. http://www.fantaproject.org/downloads/pdfs/Zambia_Guidelines_3.pdf.

[23] AKE (2000). Empfehlungen für die parentale und enterale Ernährungstherapie des Erwachsenen. Österreichische Arbeitsgemeinschaft für klinische Ernährung 3.

[24] D-A-CH (2001). Referenzwerte für die Nährstoffzufuhr. 1.Auflage. 2. korrigierter Nachdruck.

[25] Epstein L (1995). Une alimentation saine pour les personnes vivant avec le VIH/SIDA. Reseau africain des personnes vivant avec le VIH/SIDA.

[26] Arendt BM, Winkler P, Boetzer A (1999). Plasma antioxydant capacity of HIV-seropositive and seronegative subjects during long-term injection of fruit juices rich in polyphenols. Conference on Nutrition and HIV Infektion Cannes.

[27] FAO, WHO (2003). Vivre au mieux avec le VIH/SIDA: Un manuel sur les soins et le soutien nutritionnels á l’usage des personnes vivant avec le VIH/SIDA.

[28] Tchamda C, Nkabkob T (2004). Caractéristiques des ménages. Etude Démographique et de Santé du Cameroun.

[29] Epstein L (1995). Une alimentation saine pour les personnes vivant avec le VIH/SIDA. Réseau africain des personnes vivant avec le VIH/SIDA.

[30] Nguewo EA (2007). Ernährungsempfehlungen für HIV-infizierte Menschen – Entwicklung eines praktischen Leitfadens für Kamerun. Mémoire à l’université d’Albstadt-Sigmaringen.

[31] Rajabiun S, Cogill B, Seumo-Fosso E (2002). VIH/SIDA: Un guide pour les soins et le soutien nutritionnel. FANTA (Hg).

